# Ciguatoxin Occurrence in Food-Web Components of a Cuban Coral Reef Ecosystem: Risk-Assessment Implications

**DOI:** 10.3390/toxins11120722

**Published:** 2019-12-11

**Authors:** Lisbet Díaz-Asencio, Rachel J. Clausing, Mark Vandersea, Donaida Chamero-Lago, Miguel Gómez-Batista, Joan I. Hernández-Albernas, Nicolas Chomérat, Gabriel Rojas-Abrahantes, R. Wayne Litaker, Patricia Tester, Jorge Diogène, Carlos M. Alonso-Hernández, Marie-Yasmine Dechraoui Bottein

**Affiliations:** 1 Centro de Estudios Ambientales de Cienfuegos, Ciudad Nuclear, Cienfuegos 59350, Cuba; lisbet@ceac.cu (L.D.-A.); donaida@ceac.cu (D.C.-L.); miguel@ceac.cu (M.G.-B.); gabriel@ceac.cu (G.R.-A.); C.M.Alonso-Hernandez@iaea.org (C.M.A.-H.); 2 Environment Laboratories, Department of Nuclear Science and Application, International Atomic Energy Agency, 4 Quai Antoine 1er, MC 98000 Monaco, Monaco; rclausing@ucla.edu; 3 Department of Ecology and Evolutionary Biology, University of California Los Angeles, 621 Charles E Young Dr S, Los Angeles, CA 90095-1606, USA; 4 National Oceanic and Atmospheric Administration, National Ocean Service, National Centers for Coastal Ocean Science, Beaufort Laboratory, 101 Pivers Island Rd., Beaufort, NC 28516, USA; Mark.W.Vandersea@noaa.gov (M.V.); Wayne.Litaker@gmail.com (R.W.L.); 5 Refugio de Fauna Cayo Santa María, Gaviota S.A., Villa Clara 53100, Cuba; esp1.medio@plazasantamaria.gaviota.cu; 6 Ifremer, Laboratory of Environment and Resources Western Britanny, Coastal Research Unit, Place de la Croix, B.P. 40537, 29185 Concarneau CEDEX, France; nicolas.chomerat@ifremer.fr; 7 Ocean Tester, LLC, 295 Dills Point Road, Beaufort, NC 28516, USA; ocean.tester@gmail.com; 8 Marine Environmental Monitoring, IRTA, Ctra. Poble Nou km 5.5, 43540 Sant Carles de la Ràpita, Spain; jorge.diogene@irta.cat; 9 Intergovernmental Oceanographic Commission of UNESCO, IOC Science and Communication Centre on Harmful Algae, University of Copenhagen, 2100 Copenhagen, Denmark

**Keywords:** Caribbean, ciguatoxicity, qPCR, trophic transfer, ish, food safety, food security, science-based management, foodborne disease

## Abstract

In Cuba, ciguatera poisoning associated with fish consumption is the most commonly occurring non-bacterial seafood-borne illness. Risk management through fish market regulation has existed in Cuba for decades and consists of bans on selected species above a certain weight; however, the actual occurrence of ciguatoxins (CTXs) in seafood has never been verified. From this food safety risk management perspective, a study site locally known to be at risk for ciguatera was selected. Analysis of the epiphytic dinoflagellate community identified the microalga *Gambierdiscus*. *Gambierdiscus* species included six of the seven species known to be present in Cuba (*G. caribaeus*, *G. belizeanus*, *G. carpenteri*, *G. carolinianus*, *G. silvae*, and *F. ruetzleri*). CTX-like activity in invertebrates, herbivorous and carnivorous fishes were analyzed with a radioligand receptor-binding assay and, for selected samples, with the N2A cell cytotoxicity assay. CTX activity was found in 80% of the organisms sampled, with toxin values ranging from 2 to 8 ng CTX3C equivalents g^−1^ tissue. Data analysis further confirmed CTXs trophic magnification. This study constitutes the first finding of CTX-like activity in marine organisms in Cuba and in herbivorous fish in the Caribbean. Elucidating the structure–activity relationship and toxicology of CTX from the Caribbean is needed before conclusions may be drawn about risk exposure in Cuba and the wider Caribbean.

## 1. Introduction

Ciguatera poisoning caused by the consumption of fish or invertebrates contaminated with natural algal-derived ciguatoxins remains the most common non-bacterial seafood-borne disease in the world [1]. Ciguatoxins (CTXs) enter the reef food web when herbivorous fish and invertebrates consume the source organisms, benthic dinoflagellates of the genera *Gambierdiscus* and *Fukuyoa*, while grazing on macroalgae, algal turfs or on the benthos. The toxins are subsequently biotransformed, bioaccumulated, and biomagnified as they are transferred up the food chain to high trophic-level predatory fish and marine mammal species [2,3].

Although initially thought to be endemic to the Caribbean and subtropical Indo-Pacific regions, the distribution of reported cases of ciguatera has been expanding, with new, emerging endemic regions identified such as the Canary Islands and Madeira Islands and more temperate areas in Japan, New Zealand and Australia [4,5,6]. Transportation (ship ballast water discharge) together with changes in environmental conditions (i.e., construction, hurricane, land runoff) as well as global climate change (water temperature increase) are factors that drive the geographical distribution of CTX-producing dinoflagellates, with specific species thriving while others do not [7]. The growing international trade of tropical reef fish contribute to the globalization of the ciguatera problem, making it difficult to manage effectively as screening methods for detecting these toxins prior to consumption have not yet been adopted. Each year up to 25,000–50,000 cases of ciguatera are reported globally; however, the true number of cases is difficult to ascertain due to underreporting or misdiagnosis. In part, this is because no exposure marker has been found, and so clinical testing procedures are not currently available for the diagnosis, which is based entirely on symptoms and recent seafood consumption [1].

In the Caribbean region, ciguatera poisoning has been associated with carnivorous fish species belonging primarily to the families Carangidae, Lutjanidae, Sphyraenidae, Scombridae, and Serranidae [8,9]. Biotoxins, identified as Caribbean-ciguatoxins (C-CTXs) have been chemically characterized from the tissues of carnivorous species, including invertebrate feeders and benthic or pelagic predators [10,11,12,13,14,15]. The two most commonly found C-CTXs include C-CTX1 and its energetically less-favoured epimer C-CTX2 [11,16], being the proportion of C-CTX1 in these carnivores highly variable [13,17,18,19,20]. Furthermore, 10 other C-CTX analogues or isomers with different mass have also been isolated [16], but their structures have not yet been elucidated. The structure of the toxin precursors at producers (trophic level 1) in the Caribbean has not yet been confirmed, although CTX-like activity was demonstrated by biological cytotoxicity assay in species including *G. carolinianus* or *G. excentricus* and ranged from 0.02 to 469 fg CTX3C (aka P-CTX3C) equivalents per cell [21]. In contrast, marine first-order consumers (trophic level 2) of the Caribbean region have never been analyzed for their CTX contents.

Caribbean CTXs share important structural similarities to the Pacific CTXs in the central part of the molecule but differ significantly at both ends. CTX1B (aka P-CTX1B, CTX1 or P-CTX1) has 13 rings and a spiroketal at carbon-52, whereas C-CTX1 is composed of 14 rings with a hemiketal in ring N. They also differ in the number (5 for C-CTX1; 6 for CTX1B) and positions of the hydroxyl groups [9]. In a single study from 1997, the half lethal dose (LD_50_) after intra peritoneal injection of C-CTX1 in mice was found to be 3.6 µg kg^−1^ body weight (b.w.) [20], more than 10 times less toxic than CTX1B (0.25 µg kg^−1^ after Lewis et al. [10], 0.33 µg kg^−1^ after Dechraoui et al. [22], 0.35 µg kg^−1^ after Murata et al. [23], and 0.36 µg kg^−1^ after Yogi et al. [24]). Yet, competitive displacement on rat brain membrane using a receptor binding assay (RBA) gave an affinity of C-CTX1 only one-half that of CTX1B (see for example the 50% inhibitory concentration IC_50_ of 0.45 and 0.95 nM for CTX1B and C-CTX1, respectively; Pottier et al. [19]). Thus, although they present similar structural features, Caribbean and Pacific CTXs do not have the same affinities, and their relative toxicities need to be better established to assess ciguatera risk in the Caribbean.

In tropical areas, ciguatera poses a risk not only for subsistence-based fisheries and local human health, but also for fishery product exports and tourism [25]. In Cuba, as in many other endemic islands around the world, fishing for export constitutes an important part of the economy. It is valued at nearly 70 million USD and focuses mainly on species with high commercial value such as lobsters and shrimp [26]. Subsistence fishing represents a primary source of local income and food supply. As early as the middle of the 19th century, the Cuban government endeavored to mitigate ciguatera by enforcing regulation of commercial and recreational fishing [27], including size restrictions on the catch and sale of no fewer than 20 risky species (Table 1). These measures, along with the creation of the Fisheries Inspection Office and the implementation of a Hazard Analysis and Critical Control Points method in the fish-processing industry, have contributed to reduce ciguatera outbreaks in Cuba [28]. Yet, the resulting closure of highly nutritious local fisheries has forced many communities to change to less healthy and often more expensive protein sources, posing health challenges in a country where food production is limited but food security and nutrition is a key priority.

In Cuba, outbreaks of ciguatera have been reported on both the north and south coasts [29,30,31], mainly involving barracuda (*Sphyraena barracuda*) and horse-eye jacks (*Caranx latus*). Although the existence of high-risk areas is known, neither reef-collected fish nor those associated with suspected ciguatera cases have ever been tested for ciguatoxin content. In order to improve ciguatera risk assessment in the Caribbean region, this study evaluated the occurrence of ciguatoxin-like compounds in the food web in a known high-risk area of southern Cuba, including characterization of the *Gambierdiscus*/*Fukuyoa* community and toxin measurement in invertebrates and fishes.

## 2. Results

### 2.1. Benthic Dinoflagellate Composition

**Figure 1:**
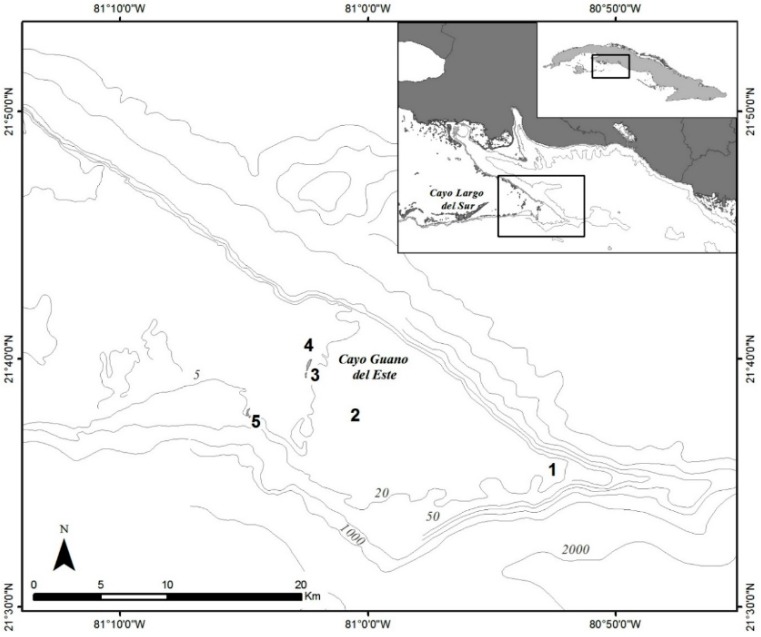
Study area on the south-central Cuba. Locations of the five sampling sites from east to west are as follows: 1: (21°34.865’ N 80°52.800’ W); 2: (21°38.229’ N 81°00.996’ W); 3: (21°39.325’ N 81°02.335’ W); 4: (21°40.220’ N 81°02.315’ W); 5: (21°37.487’ N 81°04.718’ W). The grey shaded areas indicate land.

The benthic dinoflagellate community in the study area (Figure 1) was sampled with the deployment of 20 artificial screen substrates across 5 sites, of which 18 were successfully retrieved, and the collection of four *Dictyota* samples. At two sites (4 and 5), one of the screens was not recovered, and *Dictyota* was not found at site 4.

**Figure 2:**
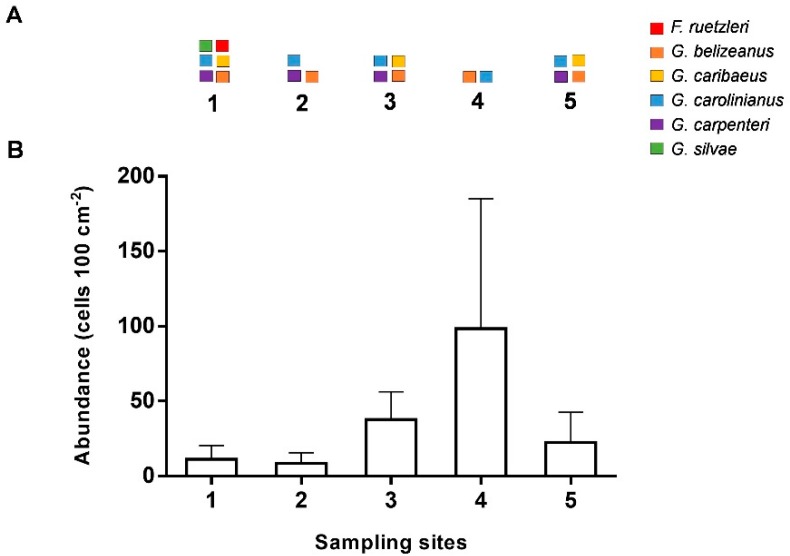
Diversity and abundance of *Gambierdiscus/Fukuyoa* spp. in field samples. (**A**): Diversity of species in screen and *Dictyota* samples using quantitative polymerase chain reaction (qPCR) assays. (**B**): Abundance (mean + standard deviation (SD)) of *Gambierdiscus*/*Fukuyoa* cells in replicate screen samples (n = 4) enumerated through microscopy.

*Gambierdiscus*/*Fukuyoa* was found to be present in all sites (Figure 2). Overall abundances (mean ± standard deviation (SD)) in screen samples were highly variable, with 33 ± 45 *Gambierdiscus*/*Fukuyoa* cells 100 cm^−2^, but were not significantly different among sites.

Quantitative polymerase chain reaction (qPCR) assays detected six species associated with screen or *Dictyota* substrates, and included *Gambierdiscus caribaeus*, *G. belizeanus*, *G. carpenteri*, *G. carolinianus*, *Fukuyoa ruetzleri*, and *G. silvae*. The species *G. carolinianus* and *G. belizeanus* were found in all sampling sites while *G. caribaeus* and *G. carpenteri* were found in three and four of the five sites, respectively. Site 4 had the highest abundance, yet the lowest diversity of *Gambierdiscus*. *Fukuyoa ruetzleri* and *G. silvae* were found only at site 1 and only on the *Dictyota* sample (Figure 2A). Species composition was highly variable among replicate screens within sites. Because qPCR estimates were at the lower end of the detection range, uncertainty prevents reporting individual values. However, these low abundances from qPCR were in correspondence with the low total visual counts (Figure 2B).

### 2.2. Identification and Toxicity of Gambierdiscus in Culture

Based on the fine structural characteristics and PCR analyses, the strain successfully established in culture (CUB5G4) was identified as *Gambierdiscus belizeanus.* The cells in culture were photosynthetic and anterioposteriorly compressed, with a depth of 68.7 ± 3.5 µm (range 61.6–75.6 µm) and width of 71.5 ± 3.5 µm (range 64.4–78.4 µm) as measured by light microscopy.

**Figure 3:**
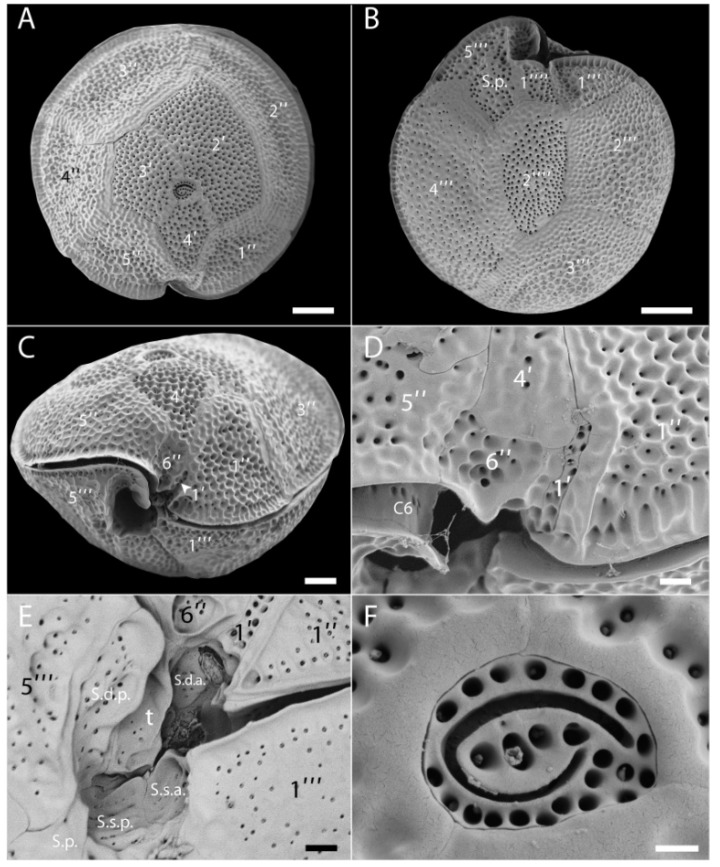
Scanning electron microscopy (SEM) micrographs of *G. belizeanus* (strain CUB5G4). (**A**): Epitheca. (**B**): Hypotheca. (**C**): Ventral view. (**D**): Detail of the small ventral plates 1′ and 6′′. (**E**): Detail of sulcal plates. (**F**): Apical pore plate (Po) with the fishhook-shaped pore. Scale bars: A, B, C: 10 µm, D, E: 2 µm and F: 1 µm.

The cells were found to have the thecal plate formula: Po, 4′, 6′′, 6C, 6S, 5′′′, 2′′′′. The epitheca comprised 11 plates (Figure 3A,D). The apical pore plate (Po) was located in a slightly ventral position (Figure 3A). It was oval in shape, and with a fishhook-shaped pore surrounded by thecal pores (Figure 3F). Plates of the apical series were very unequal in size. The first apical plate (1′) was very small and narrow, located ventrally below the lozenge-shaped 4′ and the sulcus (Figure 3C,D). The second apical plates (2′) was the largest of the series and moderately hatchet-shaped (Figure 3A). Plates 3′ and 4′ were similar in size. Among the precingular plates, the largest were the quadrangular 2′′ and pentagonal 3′′ (Figure 3A). Plates 1′′, 4′′ and 5′′ were about the same size (Figure 3A) whereas the plate 6′′ was very small and located ventrally below the 4′ plate (Figure 3C,D). The cingulum was very narrow and deep (Figure 3C); only the plate C6 at its distal end was observed (Figure 3D). The sulcus was oblique, deep and complex. From detailed observations, at least six sulcal plates could be identified (Figure 3E). Eight plates composed the hypotheca (Figure 3B). Among the postcingular plates, 1′′′ and 5′′′ were the smallest, while 2′′′, 3′′′ and 4′′′ were large (Figure 3B). The two antapical plates were very unequal in size, with 1′′′′ small and in contact with the sulcal area, while 2′′′′ was elongated, rather narrow and pentagonal (Figure 3B). The cell surface was heavily ornamented (reticulate-foveate ornamentation) and numerous thecal pores were scattered on the plates (Figure 3A–D). All these morphological features are in agreement with the original description of *G. belizeanus* by Faust [32] from Belize, Western Caribbean Sea.

**Figure 4:**
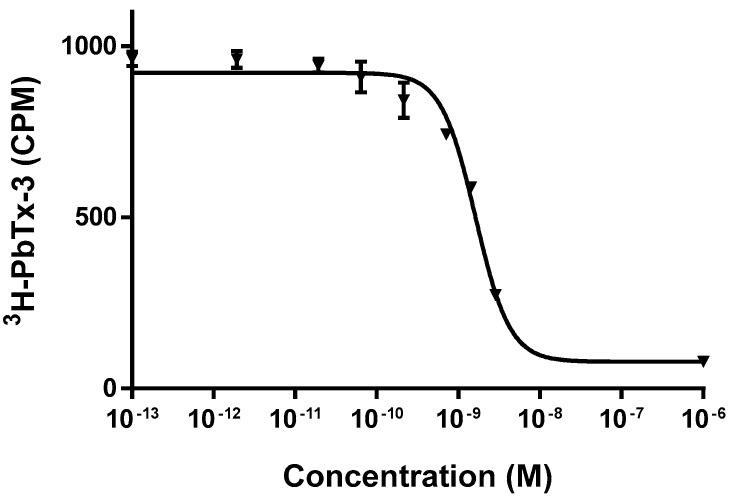
Representative dose response curve for CTX3C standard by the receptor binding assay (RBA). Each point represents the mean ± standard error (SEM) of triplicate values for a single experiment.

Ciguatoxicity of this strain was found to be below the limit of detection of the receptor binding assay, RBA (0.25 pg CTX3C equiv. cell^−1^) used. A representative CTX3C standard curve is shown in Figure 4.

### 2.3. Toxicity Assessment of Invertebrate and Fish Samples

Fish sampling encompassed a large number of species, each represented by few individuals. Seventy-five individuals of invertebrate and fish were collected, distributed within 18 distinct families, 22 genera and 28 species. Specimens were comprised of 67% carnivorous fish (n = 50), 20% herbivorous fish (n = 15), and 13% invertebrates (n = 10). Out of the 65 fish analyzed by RBA, nine were juveniles (Table 2).

**Figure 5:**
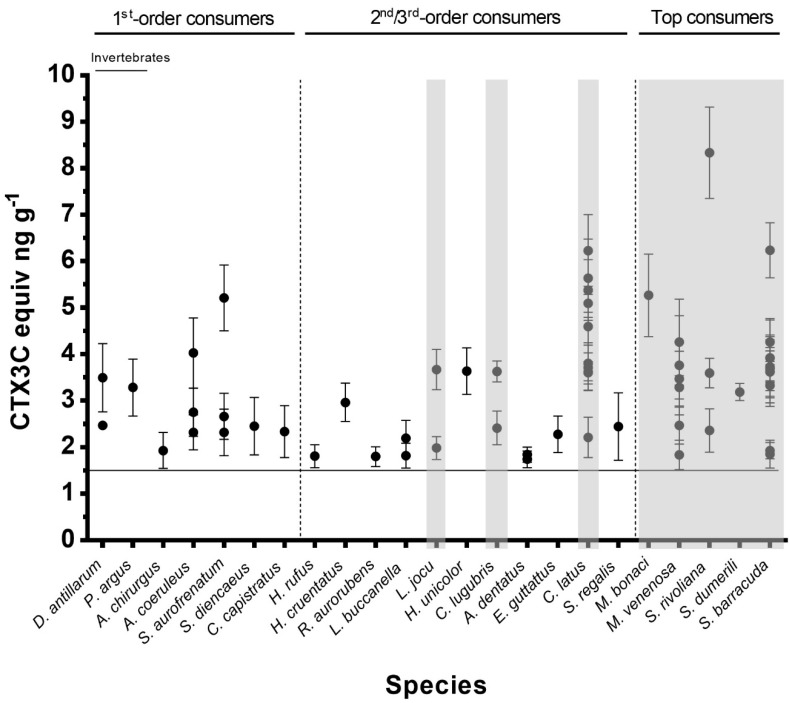
Toxicity of sampled fish and invertebrate specimens (Mean ± SD of replicate RBA analyses). Carnivorous species are arranged by increasing trophic level. The horizontal line corresponds to the RBA limit of quantification (LOQ = 1.5 ng CTX3C equiv. g^−1^). RBA^−^ specimens are not shown. Shaded areas indicate fish species banned by Cuban regulation. These data are represented in a box and whiskers plot as Supplementary Information (Figure S1). Shaded areas indicate fish species banned by Cuban regulation.

Analysis of muscle tissue by RBA (CTX3C standard curve shown in Figure 4) revealed that 80% of the samples collected in the area (59 specimens) contained levels of CTX-like activity above the RBA limit of quantification (LOQ = 1.5 ng CTX3C equiv. g^−1^ fish) and were thus classified as RBA^+^ (Table 2, Figure 5). Among the nine juvenile fish, only two were RBA^−^, one *Acanthurus chirurgus* and the sole *Sparisoma chrysopterum* (Table 2). The highest toxin concentration (8.3 ng CTX3C equiv. g^−1^) was found in a specimen of *Seriola rivoliana* (Table 2, Figure 5), which was also the largest fish collected by weight (13.65 kg). Half of the herbivorous fish were RBA^+^ (Table 2, Figure 5). The highest concentration among herbivorous fish was found in a specimen of *S. aurofrenatum* at 5.2 ng CTX3C equiv. g^−1^. Among the 10 invertebrate samples collected (Table 2), only the lobster *Panulirus argus* (n = 1) and sea urchin *D. antillarum* (n = 2) were RBA^+^; the sea urchin *Eucidaris tribuloides* (n = 1; three specimens combined), the conch *Lobatus gigas* (n = 5) and sea cucumber *Holothuria mexicana* (n = 1; Figure 5) samples were all RBA^−^.

**Figure 6:**
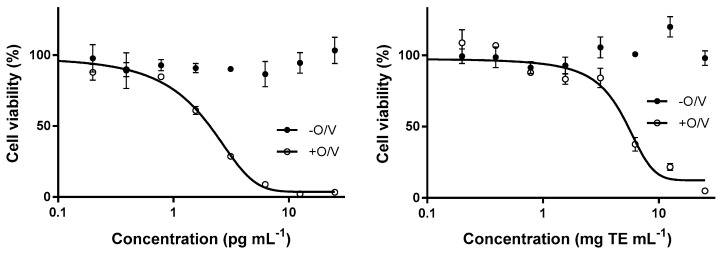
CTX1B standard curve (**left**) and a fish extract analyzed by cell-based assay (CBA-N2A, **right**). The extract illustrated here is a *Seriola rivoliana* specimen and demonstrates ciguatoxin-like activity (cytotoxicity exclusively in the presence of O/V) corresponding to an estimated 0.362 ng CTX1B equiv. g^−1^. Each point represents the mean ± SEM of triplicate values for a single experiment. O: ouabain; V: veratridine.

Results of N2A analysis of the four selected fish samples were qualitatively well-correlated with those of the RBA (i.e., RBA^+^ samples were N2A^+^ and RBA^−^ were N2A^−^). The values for the three RBA^+^ samples were 8.34, 6.24 and 1.93 estimated in ng CTX3C equiv. g^−1^ by RBA and 0.362, 0.535 and 0.0406 estimated in ng CTX1B equiv. g^−1^ by N2A. The RBA^−^ sample (LOQ: < 1.5 ng CTX3C equiv. g^−1^) was also N2A^−^ (LOQ N2A: < 0.0324 ng CTX1B equiv. g^−1^). The result of the analysis by N2A of a *Seriola rivoliana* specimen extract is represented in Figure 6.

When fish species were grouped according to their trophic level values, a significant positive correlation with toxicity was obtained, i.e., toxicity increased with increasing trophic level (rho = 0.336, *p* = 0.01). No correlations were found between body size (either weight or length) and toxicity for either *C. latus* (n = 10) or *S. barracuda* (n = 12).

## 3. Discussion

Our results confirm the ubiquitous occurrence of *Gambierdiscus* in the coastal waters of south-central Cuba, as previously reported [33]. The *Gambierdiscus* assemblage was diverse, encompassing six of the eight known *Gambierdiscus* or *Fukuyoa* species found in the Caribbean [21]. Primarily species of low toxicity were found (i.e. those reported to produce CTXs in the fg cell^−1^ range, from less than 1 fg (*G. caribeaus*) to 25 fg (*G. silvae*) CTX3C equiv. cell^−1^). More toxic species such as *G. excentricus* found in the north-eastern Gulf of Mexico or *G. polynesiensis* found in the Pacific are reported to produce toxins in the pg cell^−1^ range, at 0.47 and 5.83 CTX3C equiv. cell^−1^, respectively. Cell densities were found to be relatively low (never exceeding 100 cells 100 cm^−2^) compared to values as high as 10,000 cells 100 cm^−2^ reported in Belize [34] or 1200 cells 100 cm^−2^ in Malaysia [35]. Interpretation of sampling data with limited spatial and temporal scale, as conducted in this study (one sampling event for microalgae), requires cautious judgement considering the known geographical and temporal variability in distributions of *Gambierdiscus* spp. [35,36]. It highlights the complexity of developing an environmental monitoring strategy for seafood safety and disease management, with a need to conduct concomitant surveillance of toxins in fish for efficient monitoring.

A large majority (80%) of the invertebrate and fish from the chosen high-ciguatera risk sampling area showed CTX-like activity by RBA, with levels ranging from 1.5 to 8 ng CTX3C equiv. g^−1^. Such a high prevalence of fish contamination is not uncommon in hot spots in the Caribbean. For example, CTX was found in 74% of a sample set of commercialized species in the US Virgin Islands (USVI) [12], and in 60% of the barracudas in Marathon Key, Florida [17]. On the other hand, toxicity comparison to other studies is limited by the use of either different testing methodology or toxin reference standard used. Our data show similar toxicities to those found in Pacific fish by RBA (ranging 1 to 20 ng CTX3C equiv. g^−1^ fish) [37,38], but to our knowledge there are no studies in the Caribbean with which we can directly compare. Flesh of fish from the Virgin Islands [12] were found to contain from 0.005 to 0.18 ng C-CTX1 equiv. g^−1^, using a cytotoxicity assay CBA-N2A. In comparison, under equivalent experimental conditions, the highest concentrations (up to 2.1 ng C-CTX1 equiv. g^−1^) were found in the liver of barracudas from the Florida Keys [17]. Comparing data from different bioassays conducted using different reference toxins is challenging, and how they translate into human illness risk remains uncertain.

Even with the limited number of species and samples tested, it is worth noting that although most invertebrates were RBA^−^, the three individuals that were RBA^+^ were found with concentrations higher (mean value of 3.09 ng C-CTX1 equiv. g^−1^) than many of the fish species. Ciguatoxin occurrence in invertebrate species has been reported in other studies from the Pacific Islands [39,40], where they have been involved in ciguatera poisoning incidents [41]. However, they have not yet been included on any list of species of concern in the affected countries [42]. On the contrary, lists of high-risk fish species have been established, varying among countries, not only across oceans and seas but also within the same endemic regions [42], and none include herbivorous species in the Caribbean. Our study included those species known to be risky, but also, for the first time, herbivorous fish generally considered safe for consumption in the Caribbean, in contrast to those from CTX-endemic areas of the Pacific [8,9]. Specimens belonging to four of the five herbivorous species collected presented quantifiable levels of CTX-like activity, with concentrations reaching up to 5.4 ng CTX3C equiv. g^−1^ in their flesh. Finding significant CTX-like activity in the carnivorous fish species previously identified as risky by the Cuban government and other toxicological and epidemiological reports in the Caribbean [9,11,20,43] was expected. However, we also showed that carnivorous species large enough to be edible but not considered risky in Cuba were also RBA^+^, with values above 1.74 ng CTX3C equiv. g^−1^. An example is the blackfin snapper (*L. buccanella*), not controlled under Cuban regulations but considered as potentially toxic in other islands in the Caribbean (e.g., Saint Thomas, USVI; Olsen et al. [44]). These results demonstrate that further sampling and toxin assessment are essential in order to better understand the fate of ciguatoxin transfer up the food chain, where the main focus to date has been on a limited range of potentially toxic species.

Although CTX-like activity was detected in numerous herbivorous fish, the significant correlation between fish toxin content and trophic level indicate that predatory fishes in this region may be more toxic than those lower in the food chain. These results are in agreement with the common knowledge of ciguatera in the region. They also corroborate a study in the Pacific that found a positive correlation between CTX1B levels and δ^15^N, suggesting biomagnification may be occurring due to biotransformation of P-CTX2 and P-CTX3 precursors (aka 52-*epi*-54-deoxyCTX1B and 54-deoxyCTX1B, respectively) [45]. In the Pacific, P-CTXs are known to become more potent with metabolization and oxidation as they move up the fish food chain, resulting in a greater risk of ciguatera with large predator fish [37]. In the Caribbean, however, C-CTXs have only been identified in carnivorous fish, and it has been proposed that oxidized products of C-CTX do not lead to compounds of greater toxicity, despite their enhanced polarity [46]. While our results suggest CTX biomagnification in the Caribbean food web, the detection technique we used is based on the pharmacological properties of CTXs, and thus does not discriminate between increasing concentration and increasing binding affinity of the C-CTXs.

The two fish found to be most toxic were also the largest specimens, suggesting that cumulative bioaccumulation over time may result in higher toxicity in fish; however, this was not confirmed by relationships between weight and length with toxicity in either *C. latus* or *S. barracuda*, two predator species known to be toxic in the region. Many factors regulate tissue CTX concentrations, including species-specific rates of toxin assimilation and excretion, growth, and physiological condition, resulting in unclear relationships between body size and CTX content for most species [38,47,48,49]. Most of the juvenile fishes tested (7 of 9) showed significant toxicities at concentrations similar to adults of the same species. Moreover, the toxin quantities we measured in these juvenile fish specimens are in accordance with levels found in the muscle tissue of juvenile herbivorous fish exposed daily for two weeks to ecologically relevant dietary doses of toxic *Gambierdiscus* [50]. Taken together, these results suggest that the process of CTX bioaccumulation is not linear with size, and thus size alone may not be a reliable indicator of ciguatoxicity (see also Gaboriau et al. [38]). Therefore, the implementation of size limits on fisheries would not ensure protection against risk for ciguatera.

Major analytical challenges, including the method of analysis and the reference toxin standard used, have been highlighted for implementation of effective risk management in the Caribbean region. At present, there are no commercially available Caribbean CTX standards, and very little information exists on the chemical structure and toxicology of the analogues of CTXs present in Caribbean fish. Moreover, the lack of knowledge about the structure–activity relationship of C-CTXs precludes our ability to relate toxin measurements (e.g., RBA data) to potential exposure risk. To date, 12 congeners have been separated by mass spectrometry from Caribbean carnivorous fish, with only the C-CTX1 and C-CTX2 fully identified [16]. Toxicity has only been assessed for a purified C-CTX1, and only in a single study where its intraperitoneal LD_50_ in mice was estimated at 3.6 µg kg^−1^ b.w. [20], a value more than 10 times higher than that found for CTX1B [10,22,23,24]. This difference in potency is, in fact, the only available experimental data that support the 10-fold difference on the accepted risk exposure values between CTX1B and C-CTX1 [51]. In addition, due to the lack of available purified toxins, very few in vitro dose response bioassays (e.g., RBA, CBA-N2A) have been conducted for C-CTXs, and they have rarely been compared to those of P-CTXs, for which the structure–activity relationship is better known [22,24]. The only available direct comparison between CTX3C and C-CTX1 was made using a fluorescent RBA, where similar binding dose response curves were found for the toxins, suggesting nearly equivalent toxicities (CTX3C 1.3 times more toxic than C-CTX1) [52]. Based on the very few studies in the literature [19,24,37], RBA relative potencies between P-CTXs and C-CTXs indicate a roughly 1:1 equivalence between CTX3C and C-CTX1. Thus, it can be inferred that C-CTX1 and CTX3C would induce a similar RBA response, indicating that our data, expressed in CTX3C equivalents, would have equivalent values if C-CTX1 standard had been used. However, extrapolating this value to potential risk exposure presents challenges, where other toxin congeners can exhibit different intrinsic behaviour than C-CTX1. This may be particularly true for samples such as herbivorous fish, which contain numerous toxin metabolites. Other interfering metabolites include the gambieric acid D, a compound capable of binding to the voltage gated sodium channels but with much less affinity than CTXs [53]. These molecules are produced by *Gambierdiscus* species and were recently found in shark flesh [54].

The analysis of fish remnants associated to ciguatera cases in the French West Indies have provided some insight into toxin intake and ciguatera symptoms [8]. In this study mouse bioassay pre-screened fish samples were analyzed against a reference standard from the Pacific (CTX1B) on the CBA-N2A, and a lowest observable adverse effects level (LOAEL) was estimated at 4.2 ng CTX1B equiv. individual^−1^ corresponding to 48.4 pg CTX1B equiv. kg^−1^ b.w. The absence of a correlation between the mouse bioassay and the CBA-N2A noted in the study, as well as the significant difference in the slope of the dose response curve on the CBA-N2A assay of the C-CTX in the fish matrix compared to that of the CTX1B, again raise the need for better characterization of the C-CTX structure–activity relationship and toxicology.

In conclusion, analysis of samples from a reef known to be at risk for ciguatera on the south-central Cuba revealed low abundances of widely distributed *Gambierdiscus* species and a trophic food web contaminated with CTXs from lower trophic levels to top predators. Slightly higher toxicity in upper trophic level fish suggested biomagnification up the food chain. However, the finding of numerous RBA^+^ herbivorous fish species indicates these herbivores could also serve as a vector for human intoxication, despite the lack of reported ciguatera cases following the consumption of herbivorous fish in Cuba. Finally, the measurement of toxin values useful for risk assessment remains challenged by the lack of appropriate CTX standards and knowledge of structure–activity relationships of Caribbean CTXs. Elucidating the toxicology of C-CTXs and determining toxic equivalent factors between all known CTXs using fully validated methods of analysis remain essential to support the development and implementation of effective ciguatera monitoring and management programs.

## 4. Materials and Methods

In December 2016, we sampled a reef off the coast of south-central Cuba in Puntalón, a bank known by local residents to be unsafe due to risk of ciguatera (Figure 1). The nearest cay to this area is Cayo Guano del Este. Other cays are found successively to the west toward Cayo Largo del Sur, a small resort island where coral reefs constitute one of the main attractions for tourists. Five sampling sites were selected to encompass depths between 5 to 10 m (sites 2, 3 and 4) or up to 20 m (sites 1 and 5) (Figure 1) to increase the probability to detect different *Gambierdiscus* species, particularly those that may be toxic [55].

All five sampling sites were generally characterized by a hard bottom with patch reefs and abundant macroalgae. The deep sites (1 and 5) were composed of reef ridges covered by corals, gorgonians and abundant macroalgae, while sites 2 and 3, both at 10 m depth, were hard bottom with less relief. Corals, gorgonians, and macroalgae such as *Dictyota* and *Sargassum* were predominant in both, but site 3 had higher algal cover as well as a higher abundance of herbivorous fish. At 5 m depth, site 4 was highly dynamic with a flat bottom containing few corals and smaller-sized gorgonians.

### 4.1. Sample Collection

At all sampling sites, the *Gambierdiscus*/*Fukuyoa* community was sampled using artificial substrates (fiberglass screen 17 cm × 11 cm; n = 4) deployed for 24 h. The sampling methodology and the processing of samples followed that described by Tester et al. [34]. Where present, single samples (one per site) of *Dictyota* were also collected and processed similarly. Briefly, each screen was attached to monofilament fishing line and suspended in the water column approximately 10 cm from the bottom using a weight and small subsurface float. Screens were retrieved on scuba diving by gently placing in individual 900 mL plastic jars while underwater. The jars were sealed with a screw cap and returned to the surface. The jars were then shaken vigorously on the boat to dislodge epiphytic dinoflagellates from the screens and poured through a 200 µm sieve into a graduated cylinder from which the total volume was recorded. For screen samples, the sieved seawater was filtered again through a 20 µm pore size nylon mesh to collect the benthic dinoflagellate cells. For *Dictyota* samples, roughly 100–150 mL of the sieved seawater was filtered with 20 µm mesh and the exact volume recorded. In both cases, the nylon mesh was then transferred to a 50 mL screw-cap tube containing 50 mL of GF-1 (Macherey-Nagel GmbH and Co. KG, Düren, Germany) filtered seawater and 2 drops of neutral Lugol’s iodine solution. The remaining sieved seawater from *Dictyota* samples was transported in clear bottles without preservation to the laboratory and kept in a culture chamber until isolation of cells.

Fish were collected, via spear guns or hook and line, throughout the sampling area during five sampling events between December 2016 and January 2018. Invertebrates were collected by hand on scuba. We targeted herbivorous fish species commonly found in the area, including parrotfish, surgeonfish and tangs, as well as carnivorous fish species known to cause ciguatera (Table 1). Whenever possible, several individuals per species were collected in order to screen the widest range of sizes and weight possible. Total length (to the nearest cm) and weight (to the nearest g) were recorded and used to classify each specimen as juvenile or adult following species-specific thresholds for sexual maturity given in FishBase [56]. The fish were transported on ice to the laboratory where they were identified to species level using the morphological features described in the Food and Agriculture Organization (FAO) species identification guide for fishery purposes [57]. Then they were filleted and stored at −20 °C until CTX extraction.

### 4.2. Gambierdiscus/Fukuyoa Identification and Counting

For each sample, the genera *Gambierdiscus*/*Fukuyoa* were identified using a Zeiss Axiovert 40 inverted microscope at 100× magnification. Preserved samples were shaken to suspend benthic dinoflagellate cells homogenously, from which three to four 1 mL aliquots were taken from each sample and counted using a Sedgewick Rafter slide. The average abundance of each sample was calculated using the appropriate volumetric conversion factors and the effective area of the screen [34]. Average abundances were expressed as cells 100 cm^−2^ or as cells g^−1^ wet weight of *Dictyota*.

### 4.3. Molecular Identification of Gambierdiscus and Fukuyoa Species in Field Samples

Semi-quantitative, species-specific qPCR assays were performed at the National Oceanic and Atmospheric Administration, National Center for Coastal Ocean Science Laboratory in Beaufort, North Carolina USA to survey the microalgal samples for relative cell abundance and *Gambierdiscus* species distribution. Each aliquot was filtered onto 47 mm diameter, 8 µm pore-size polycarbonate filters. DNA was extracted from each filter as described by Vandersea et al. [58] using the Mo Bio Power Soil DNA isolation Kit (Carlsbad, CA, USA) following the manufacturer’s protocol except that 350 µL of cell lysate rather than the prescribed 450 µL was processed. The DNA extracts were eluted from the mini columns using 50 µL of elution buffer and stored at 4°C. DNA was screened for the presence of *G. belizeanus*, *G. caribaeus*, *G. carolinianus*, *G. carpenteri*, *F. ruetzleri*, *G. silvae*, *G. excentricus*, and *Gambierdiscus* sp. ribotype 2 using the species-specific PCR primers described in Vandersea et al. [58] and Litaker et al. [59]. The samples were also screened for the presence of *G. australes*, *G. pacificus*, *G. polynesiensis*, and *G. toxicus*, which served as negative controls since these taxa have not yet been found in the Caribbean region.

qPCR assays were performed using an Eppendorf Mastercycler^®^ ep RealPlex 4 system with white Eppendorf real-time tube strips (Eppendorf North America, Inc., Westbury, New York, NY, USA) and a total reaction volume of 10.5 µL per tube. Each PCR reaction mixture contained 4.5 µL of 5 Prime RealMasterMix SYBR ROX 2.5X (0.05 units µL^−1^ Taq DNA polymerase, 10 mM Mg (CH3COO)_2_, 1.0 mM dNTPs, 20X SYBR^®^ Green solution), each primer at a concentration of 0.15 µM, 4.7 µL of sterile deionized water and 1 µL of template DNA. Thermal cycling conditions included denaturation at 95 °C for 2 min followed by 40 cycles at 95 °C for 10 s, annealing at 58 °C for 15 s with a subsequent extension at 68 °C for 20 s. The fluorescence threshold was determined by the Eppendorf RealPlex 4 analytical software, and the PCR cycle during which fluorescence crossed the threshold was designated the quantification cycle (Cq). A melting curve analysis was performed following thermal cycling to check the specificity of the PCR reactions. The melting curve profile consisted of denaturation at 95 °C for 15 s followed by an annealing step for 15 s at 58 °C. The fluorescence was continuously monitored during a continuous 20-min temperature ramp from 58–95 °C, which was held at 95 °C for 15 s. The melting curve analysis was conducted by comparing the melting temperature peak of positive control DNA to other experimental DNA samples. A limit of ± 0.5 °C for melting temperature peak shift was set as the cutoff for species-specific amplifications. qPCR assay controls included a positive control, a no template control, and a blank extraction control to test for possible DNA cross contamination during the DNA extraction procedures.

### 4.4. Gambierdiscus Isolation and Culture

Individual cells were isolated from field samples using a glass Pasteur pipette drawn to a narrow filament with the aid of a Zeiss Axiovert 35 inverted microscope (Zeiss, Jena, Germany). Approximately 40 cells from each site were sequentially transferred through five drops of seawater taken from the original sample and sterilized by filtration before being transferred into individual wells of a 96-well plate containing 50% of f10K culture media [60] in sterile seawater. The plates were incubated at 26 °C with a 12:12 day/night photoperiod. Once the cells completed several division cycles, they were transferred to a 24-well culture plate containing f10K culture media and subsequently to 50 mL glass Erlenmeyer flasks for further growth. Cultures in the exponential growth phase were then pelleted using centrifugation from a known volume and cell density and stored at 20 °C until extraction.

#### Morphological Identification

From the *Dictyota* samples taken, one strain was successfully established in culture from the site 3 sample. Cells were fixed in Lugol’s iodine solution at a final concentration of 4%, and 30 cells were randomly selected and observed under an inverted microscope (Axiovert 40 CFL, Zeiss, Jena, Germany). The depth (dorso/ventral distance) and width (transdiameter) of each cell were measured at 400× magnification. For SEM examination, fixed cells were rinsed in distilled water and then isolated with a micropipette. Isolated cells were placed on a polycarbonate filter (1.2 µm pore size), dehydrated in ethanol baths of increasing concentration, and critical-point dried in a EMS850 CPD (Quorum Technologies, Ashford, UK) according to Chomérat and Couté [61]. The filters were then coated with gold using a Cressington 108Auto (Cressington, Watford, UK) sputter coater. Cells were observed with a Zeiss SIGMA 300 (Zeiss, Jena, Germany) field-emission Scanning Electron Microscope (1.5 kV). The Besada’s tabulation system was adopted to name the thecal plates [62].

### 4.5. Ciguatoxin Analysis in Cell and Fish Samples

#### 4.5.1. Toxin Extraction

Cell extracts were prepared from *Gambierdiscus* cultures following the procedure described in Chinain et al. [63] with some modifications [33]. Briefly, cell pellets were pulse sonicated twice in absolute methanol (MeOH) and twice in aqueous methanol (MeOH:H_2_O 50:50). After adjusting the combined volumes to 60% aqueous MeOH, a solvent: solvent (1:1) partition with dichloromethane (DCM) was used to isolate the CTXs. The organic, CTX-containing DCM phase was dried under nitrogen flux, resuspended in MeOH, and stored at –20°C until analysis.

Fish samples were extracted following the procedure described by Díaz-Asencio et al. [64] based on the protocols by Lewis et al. [10] and Satake et al. [65]. Briefly, tissue samples (3–5 g) were cooked in a water bath at 70 °C for 15 min and homogenized in acetone to extract soluble compounds. After centrifugation and drying acetone supernatants, two solvent/solvent partitions with hexane/90% aqueous MeOH and DCM/60% aqueous MeOH (1:1 v:v) were applied to remove lipids and separate CTXs from other concomitant toxins (e.g., maitotoxins), respectively. The resulting DCM extract was dried and resuspended in MeOH to 10 g tissue equivalent (TE) mL^−1^ and stored at –20 °C until further analysis. Toxin recovery from fish samples was estimated to be around 50% (Rañada et al., in preparation).

#### 4.5.2. Toxin Measurement Using a Radioligand-Receptor Binding Assay

Toxicity of cell and fish extracts was assessed with a radioligand-receptor binding assay (RBA) in microplate format according to IAEA-TECDOC-1729 [66] with modifications following Dechraoui Bottein and Clausing [67] and as verified in Díaz-Asencio et al. [64]. In this assay, the radiolabeled toxin (here ^3^H-PbTx-3) competes with unlabeled molecules from the sample or standards for a finite number of available receptor sites provided by a brain membrane preparation. Dilutions of standards of CTX3C (Wako-Pure Chemicals, Osaka, Japan), a quality control (QC) of brevetoxin (Sigma Aldrich, St. Louis, MO, USA), working solutions of the tritiated brevetoxin (American Radiolabeled Chemicals Inc., St Louis, USA), and of porcine brain membrane homogenate (Sigma Aldrich, St. Louis, MO, USA) were prepared as in Díaz-Asencio et al. [64]. Dilutions of *Gambierdiscus* extracts (equivalents to 7500, 5000, 2500 and 1250 cells mL-1 in well) and of fish extracts (0.3 and 0.6 g TE mL^−1^ in well) were tested to encompass the expected range of potential toxin concentrations within known limits of detection. Previous experiments in control fish showed no matrix effect at the highest dose tested [50,64]. Stock samples for *Gambierdiscus* (~85,700 cells mL^−1^) and for the fish extracts (10 g TE mL^−1^) were dried under nitrogen flux and resuspended in buffer to avoid methanol in the assay.

To perform the assay, the filter membranes of the microplate wells (96-well MultiScreen HTS FB Filter Plate MSFBN6B50, Millipore) were first moistened with 35µL of Phosphate Buffered Saline Tween 20 (pH 7.4, Sigma Aldrich, St. Louis, MO, USA) buffer amended with bovine serum albumin (1 g L^−1^). CTX3C standards, QC check or dilutions of cells or fish extracts (35 µL) were then added to the corresponding wells, followed by 35 µL of 3H-PbTx-3 working solution (8.72 nM). Finally, 195 µL of brain membrane homogenate was added to each well, and the plate was incubated for 1 h at 4°C before filtration and rinsing on a MultiScreen HTS vacuum manifold system. After removing the plastic bottom, blotting the filter, and placing the microplate in a counting cassette, 50 µL of scintillation cocktail were added to each well. The cassette with the microplate was dark-incubated at room temperature for 2 h before counting activity in a microplate liquid scintillation counter (Microbeta^2^, PerkinElmer, Massachusetts, USA). At least two dilutions of each fish or cell extract (n = 1 for each individual sample) were tested in triplicate in three independent assays.

RBA data were analyzed as per IAEA-TECDOC-1729 [66] and Díaz-Asencio et al. [64] with GraphPad Prism software version 6 (GraphPad, San Diego, CA, USA). Sample concentrations were expressed as CTX3C equivalents, according to the toxin standard used in the assay. Toxicity results were reported as mean concentration ± SD from the three independent assays, where each assay value was calculated as the mean across triplicates of the dilutions.

#### 4.5.3. Toxin Measurement Using a Cell-Based Assay (CBA-N2A)

RBA results of fish samples were verified with a functional cytotoxicity assay using neuroblastoma N2A cells (cell-based assay (CBA)-N2A) with CTX1B as the standard at the Marine Environmental Monitoring, IRTA, Sant Carles de la Ràpita, Spain. Four fish extracts representing a maximal range of toxicity were used: the two most toxic samples, a marginally toxic sample, and one below the limit of detection of RBA.

The CBA-N2A followed a modified procedure developed by Manger et al. [68] based on ouabain and veratridine exposure and detection with the MTT [3-(4,5-dimethylthiazol-2-yl)-2,5-diphenyltetrazolium] colorimetric assay. Before starting the assay, N2A cells (cell line CCL-131, American Type Culture Collection) were plated (35,000 cells per well) on Roswell Park Memorial Institute (RPMI) medium + 5% fetal bovine serum for 24 h to reach about 70% confluence. Cell were then pre-treated with ouabain (0.1 mM Sigma-Aldrich)/veratridine (0.01 mM, Sigma-Aldrich), and exposed to either CTX1B standard (8 dilutions between 0.4 to 50 pg mL^−1^ in assay wells, provided by Richard Lewis, The Queensland University, Queensland, Australia) or fish sample extracts in culture medium [8]. After 24 h incubation, cell viability was assessed using MTT salt. Sodium channel-dependent toxic activity was assessed to ensure specificity of the assay by additionally testing each sample in the absence of ouabain (O)/veratridine (V). Prior to addition to N2A cells, methanol was removed from the CTX1B standards (or fish extracts) by evaporation under nitrogen flux and re-dissolution in RPMI medium [69].

Dose response curves with CTX1B standard were performed daily, before testing the samples, in order to evaluate cell response and define the limit of quantification (IC_20_) according to the status of the cells. Assays were repeated a minimum of three-times, with each run performed in duplicate. Data were analyzed with GraphPad Prism software v6. Viability of cells were expressed in relation to the viability of the corresponding cell control (+O/V, −O/V). The IC_50_ of the + O/V treatment was calculated using sigmoidal regression curve with variable Hill slope. Sample concentrations were expressed as CTX1B equivalents.

### 4.6. Data Analysis

Differences in abundances of *Gambierdiscus* among sampling sites were analyzed with a non-parametric Kruskal-Wallis test and *a posteriori* mean comparisons (*p* < 0.05) using STATISTICA v12.001. Potential trophic biomagnification was examined using trophic levels of sampled fish species extracted from FishBase. Relationships between trophic level and toxin levels were tested with a non-parametric Spearman rank correlation test, as data did not meet assumptions of normality. Further, within two carnivorous species well-known to be ciguatoxic, *Sphyraena barracuda* and *Caranx latus*, we evaluated the existence of relationships between toxicity and weight or between toxicity and length using Pearson or Spearman rank correlations, depending on the shape of the error distributions of the data. All analyses were preceded by tests of assumptions and removal of outliers.

## Figures and Tables

**Table 1:** Fish regulation in Cuba.

Scientific Name	Common Name	Local Name	Family	Weight (#)
*Mycteroperca bonaci*	Black grouper	Aguají o bonací arará	Serranidae	(1)
*Mycteroperca venenosa*	Yellowfin grouper	Arigua o bonací cardenal	Serranidae	(2)
*Mycteroperca tigris*	Tiger grouper	Bonací gato	Serranidae	(3)
*Carangoides bartholomaei*	Yellowjack	Cibí amarillo	Carangidae	(4)
*Seriola rivoliana*	Longfin yellowtail	Coronado	Carangidae	(3)
*Seriola zonata*	Banded rudderfish	Coronado de bandas	Carangidae	(3)
*Seriola dumerili*	Greater amberjack	Coronado de ley	Carangidae	(3)
*Lutjanus cyanopterus*	Cubera snapper	Cubera	Lutjanidae	(5)
*Caranx latus*	Horse-eyejack	Gallego o jurel	Carangidae	(6)
*Chilomycterus reticulatus*	Spotfin burrfish	Guanábana	Diodontidae	(3)
*Rypticus saponaceus*	Greater soapfish	Jaboncillo o jabón	Grammistidae	(3)
*Lutjanus jocu*	Dog snapper	Pargo jocú	Lutjanidae	(4)
*Gymnothorax funebris*	Green moray	Morena verde	Muraenidae	(3)
*Ogcocephalus vespertilio*	Seadevil	Pez diablo	Ogcocephalidae	(3)
*Diodon holocanthus*	Longspined porcupinefish	Pez erizo	Diodontidae	(3)
*Sphyraena barracuda*	Great barracuda	Picúa o Picuda	Sphyraenidae	(3)
*Diodon hystrix*	Spot-fin porcupinefish	Puerco espín	Diodontidae	(3)
*Lagocephalus laevigatus*	Smooth puffer	Tamboril gigante	Tetrodontidae	(3)
*Sphoeroides testudineus*	Checkered puffer	Tamboril rayado	Tetrodontidae	(3)
*Caranx lugubris*	Black jack	Tiñosa o Tiñosa prieta	Carangidae	(3)

(#) indicate restricted weight (1) > 4.5 Kg; (2) > 4.6 Kg; (3) any weight; (4) > 1.4 Kg; (5) > 6.8 Kg; (6) > 1 Kg.

**Table 2:** Characteristics and toxicity of fish and invertebrate species collected in the sampling area.

Species	Family	Trophic Level	Weight (range) g	Total Length (range) cm	N	RBA^+^	RBA^−^
**Invertebrates**							
*Diadema antillarum*	Diadematidae				2	2	0
Eucidaris tribuloides*	Cidaridae				1	0	1
*Holothuria mexicana*	Holothuriidae				1	0	1
*Lobatus gigas*	Strombidae				5	0	5
*Panulirus argus*	Palinuridae				1	1	0
**Herbivorous fishes**							
*Acanthurus chirurgus*	Acanthuridae	2	86–220	17–21	6	1	5 (1)
*Acanthurus coeruleus*	Acanthuridae	2	150–310	19–23	4	3	1
*Sparisoma aurofrenatum*	Scaridae	2	147–165	20–21	3	3	0
*Sparisoma chrysopterum*	Scaridae	2	180	21	1	0	1 (1)
*Stegastes diencaeus*	Pomacentridae	2	22	11	1	1	0
**Carnivorous fishes**							
*Apsilus dentatus*	Lutjanidae	4.08	950–1615	40–48	2	2	0
*Caranx latus*	Carangidae	4.16	1355–2200	49–59	10	10	0
*Caranx lugubris*	Carangidae	4.0	3195–3505	66–68	3	2	1
*Chaetodon capistratus*	Chaetodontidae	3.43	13	8	1	1	0
*Epinephelus guttattus*	Serranidae	4.15	287	27	1	1	0
*Heteropriacanthus cruentatus*	Priacanthidae	3.7	113	19	1	1	0
*Holocentrus rufus*	Holocentridae	3.49	69	21	1	1	0
*Hypoplectrus unicolor*	Serranidae	3.97	12	9	1	1	0
*Lutjanus buccanella*	Lutjanidae	3.9	1025–1170	41–43	2	2	0
*Lutjanus jocu*	Lutjanidae	3.92	3350–5250	58–62	2	2	0
*Mycteroperca bonaci*	Serranidae	4.5	2000	55	1	1 (1)	0
*Mycteroperca venenosa*	Serranidae	4.5	753–9400	37–89	6	6 (2)	0
*Pterois volitans*	Scorpaenidae	3.44	234	25	1	0	1
*Rhomboplites aurorubens*	Lutjanidae	3.8	685	37	1	1	0
*Scomberomorus regalis*	Scombridae	4.38	636	51	1	1 (1)	0
*Seriola rivoliana*	Carangidae	4.5	1795–13650	54–111	3	3 (2)	0
*Seriola dumerili*	Carangidae	4.5	14000	110	1	1	0
*Sphyraena barracuda*	Sphyraenidae	4.5	785–6400	56–112	12	12 (1)	0

RBA^+^ and RBA^−^ indicate values above and below limit of quantification (LOQ) of the receptor binding assay (RBA), respectively. Values in parenthesis indicate the number of juveniles. Shaded areas indicate fish species banned by Cuban regulation. * Three specimens combined.

## Supplementary Materials

The following are available online at https://www.mdpi.com/2072-6651/11/12/722/s1: Figure S1: Toxicity of sampled fish and invertebrate specimens. Horizontal bars represent median RBA analysis among specimens, boxes extend from the 25th to 75th percentiles and whiskers represent min to max specimens RBA values. The horizontal line corresponds to the RBA limit of quantification (LOQ = 1.5 ng CTX3C equiv. g-1). RBA- specimens are not shown. Shaded areas indicate fish species banned by Cuban regulation.
