# Supplementary material for: Ciguatoxin Occurrence in Food-Web Components of a Cuban Coral Reef Ecosystem: Risk-Assessment Implications
Source: Toxins (Basel). 2019 Dec 11;11(12):722. doi: 10.3390/toxins11120722 (PMC6950047; doi:10.3390/toxins11120722)
Supplement: Supplementary file 1 [file toxins-11-00722-s001.pdf]

Supplementary Materials: Ciguatoxin Occurrence in Food Web Components of a Cuban Coral Reef Ecosystem: Risk Assessment Implications

Lisbet Díaz-Asencio, Rachel J. Clausing, Mark Vandersea and Donaida Chamero-Lago

Miguel Gómez-Batista, Joan I. Hernández-Albernas, Nicolas Chomérat, Gabriel Rojas-Abrahantes,

Wayne Litaker, Patricia Tester, Jorge Diogène and Carlos Alonso-Hernández and

Marie-Yasmine Dechraoui Bottein

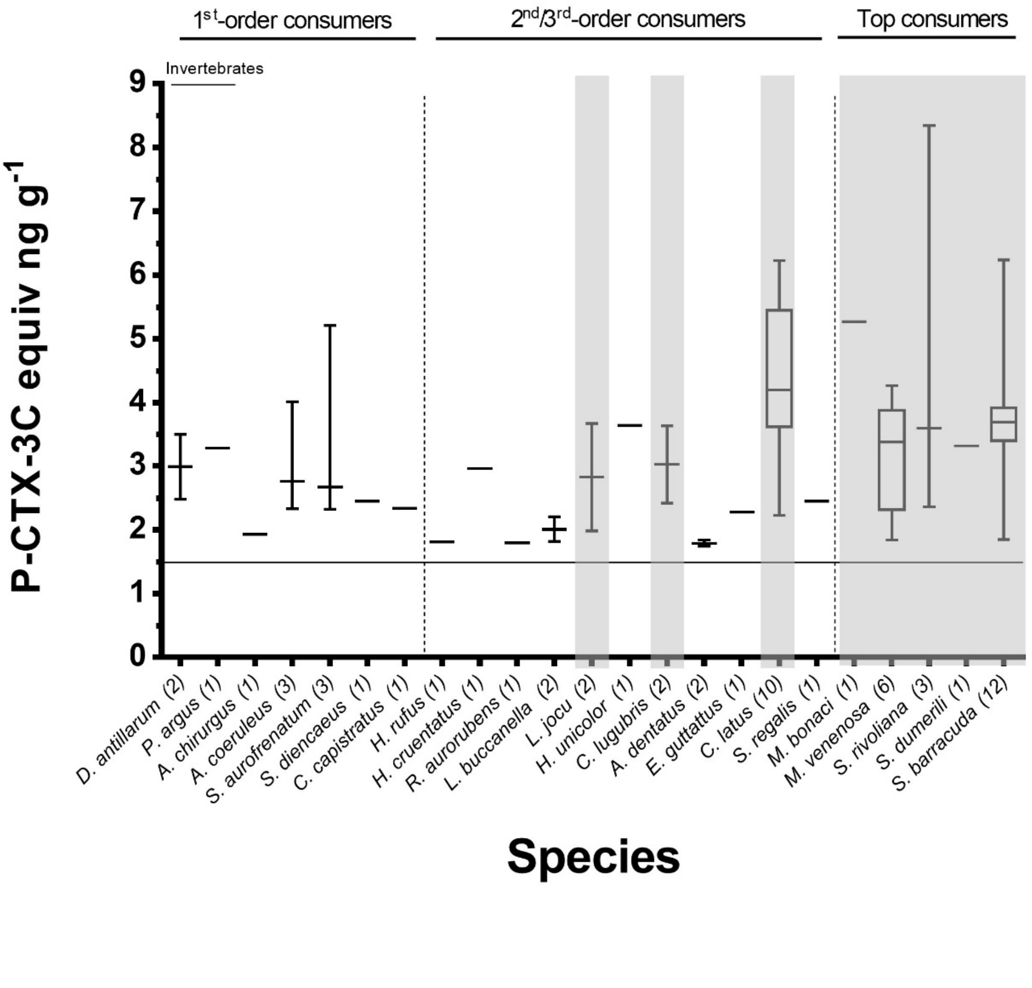

Figure S1. Toxicity of sampled fish and invertebrate specimens. Horizontal bars represent median RBA analysis among specimens, boxes extend from the 25th to 75th percentiles and whiskers represent min to max specimens RBA values. The horizontal line corresponds to the RBA limit of quantification (LOQ =1.5 ng P-CTX-3C equiv. g^{-1}). RBA^{-} specimens are not shown. Shaded areas indicate fish species banned by Cuban regulation.
